# Benchmark-Driven Clinical Decision Framework for Multi-Class Middle Ear Disease Diagnosis: Superiority of Swin Transformer in Accuracy and Stability

**DOI:** 10.3390/diagnostics16030482

**Published:** 2026-02-05

**Authors:** Guoping Chen, Haoyi Zhang, Junbo Zeng, Yuexin Cai, Dong Huang, Yubin Chen, Peng Li, Yiqing Zheng

**Affiliations:** 1 The First Clinical Medical College, Jinan University, Guangzhou 510632, China; 2 Department of Otolaryngology-Head and Neck Surgery, Zhongshan People’s Hospital, Zhongshan 528403, China; 3 College of Mathematics and Information, South China Agricultural University, Guangzhou 510642, China; 4 Department of Otolaryngology, Sun Yat-Sen Memorial Hospital, Sun Yat-Sen University, 107 Yanjiang West Road, Guangzhou 510120, China; 5 Department of Otolaryngology Head and Neck Surgery, The Third Affiliated Hospital, Sun Yat-Sen University, Guangzhou 510630, China; 6 Shenshan Medical Center, Sun Yat-Sen Memorial Hospital, Sun Yat-Sen University, Shanwei 518000, China

**Keywords:** artificial intelligence, deep learning, Swin Transformer, computer-aided diagnosis, otitis media, tympanic membrane

## Abstract

**Background/Objectives:** The variable accuracy of middle ear disease diagnosis based on oto-endoscopy underscores the need for improved decision support. Although convolutional Neural Networks (CNNs) are currently a mainstay of computer-aided diagnosis (CAD), their constraints in global feature integration persist. We therefore systematically benchmarked state-of-the-art CNNs and Transformers to establish a performance baseline. Beyond this benchmark, our primary contribution is the development of a probability-guided Top-K clinical decision framework that balances high accuracy with complete case coverage for practical deployment. **Methods:** Using a multicenter dataset of 6361 images (five categories), we implemented a two-stage validation strategy (fixed-split followed by 5-fold cross-validation). A comprehensive comparison was performed among leading CNNs and Transformer variants assessed by accuracy and Macro-F1 score. **Results:** The Swin Transformer model demonstrated superior performance, achieving an accuracy of 95.53% and a Macro-F1 score of 93.37%. It exhibited exceptional stability (95.61% ± 0.38% in cross-validation) and inherent robustness to class imbalance. A probability-guided Top-2 decision framework was developed, achieving 93.25% accuracy with 100% case coverage. **Conclusions:** This rigorous benchmark established Swin Transformer as the most effective architecture. Consequently, this study delivers not only a performance benchmark but also a clinically actionable decision-support framework, thereby facilitating the deployment of AI-assisted diagnosis for chronic middle ear conditions in specialist otology.

## 1. Introduction

Otitis media and related middle ear diseases represent a global health challenge, being some of the most prevalent and disabling diseases, especially in children [1,2,3]. In low- and middle-income countries, the prevalence of chronic suppurative otitis media reaches 1–6%, leading to significant morbidity, including hearing loss and severe complications [2,4]. Accurate and timely diagnosis is crucial for preventing these outcomes [5]. However, diagnosis remains challenging, with primary care physicians achieving only 50–70% accuracy based on oto-endoscopic tympanic membrane (TM) images [6,7,8], with even specialists demonstrating an average accuracy of 72.3% [9]. This diagnostic gap, exacerbated by global disparities in specialist access [3], underscores the urgent need for standardized, objective computer-aided diagnostic (CAD) tools.

The advent of deep learning, particularly Convolutional Neural Networks (CNNs), marked a significant improvement over traditional feature-based methods, establishing CNNs as the dominant paradigm for automated TM image analysis [10,11,12,13,14]. Models like VGG and ResNet have demonstrated accuracies exceeding 90% in controlled settings. Despite their success, CNN-based approaches face inherent limitations that constrain their clinical applicability: (1) their local receptive fields hinder effective global context modeling, which is crucial for complex medical images; (2) their performance can plateau when confronted with complex or atypical cases [15,16]; and (3) their limited interpretability remains a barrier to building clinical trust [17,18]. These bottlenecks necessitate architectural innovations that can overcome these fundamental constraints.

Therefore, it is imperative to explore next-generation deep learning architectures that possess a stronger capacity for capturing global contextual information and intrinsic interpretability. In recent years, Vision Transformer models, with powerful global self-attention mechanisms, have demonstrated the potential to achieve competitive performance surpassing that of CNNs across various computer vision domains [19,20,21,22,23,24,25]. However, their potential for multi-class oto-endoscopic image diagnosis remains underexplored in a rigorous, benchmarked manner. The relative performance, robustness, and clinical utility of these emerging architectures (including ViT, DeiT, Swin Transformer, among others) for complex medical image diagnostic tasks—particularly in multi-class tympanic membrane image analysis—also remain underexplored and lack systematic benchmark comparisons.

To address this research gap, this study aims to conduct a comprehensive benchmark study to systematically evaluate and compare the performance of leading deep-learning models in multi-class tympanic membrane image diagnosis. Rather than pre-specifying an optimal model, we are committed to identifying the most reliable solution from mainstream architectures through a rigorous experimental design. Our specific objectives are as follows:

1. To establish an accurate performance benchmark on a standardized, multicenter dataset comprising five diagnostic categories.
2. To perform an extensive and fair comparison between representative CNN models (e.g., VGG16, ResNet50, Inception-v3) and Transformer variants (e.g., ViT, DeiT, Swin Transformer) under consistent experimental conditions to determine their relative performance.
3. To conduct an in-depth analysis of the optimal model’s robustness to class imbalance and, based on its output characteristics, develop a practical, probability-guided Top-K clinical decision-support framework to provide a solution for real-world applications.

## 2. Materials and Methods

### 2.1. Dataset Construction

#### 2.1.1. Data Source and Collection

**Figure 1:**
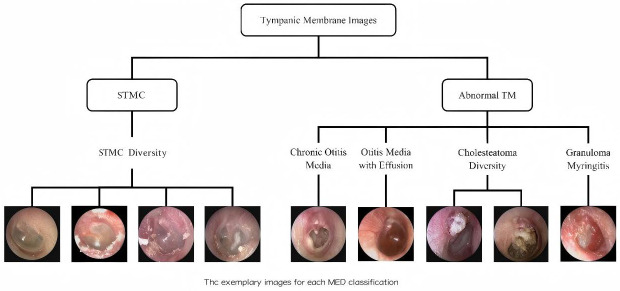
Schematic diagram of tympanic membrane image classification. The images were categorized into ‘STMC’ and ‘Abnormal TM’ branches. The ‘STMC’ section showcases the diversity of STMC images, while the ‘Abnormal TM’ section was subdivided into four categories with representative images. Abbreviations: TM, Tympanic Membrane, STMC: Stable Tympanic Membrane Condition, MED: Middle Ear Condition.

A retrospective analysis was conducted on data from 8283 patients across three tertiary hospitals (2015–2019). Tympanic membrane (TM) imaging was standardized using 2.7 mm rigid endoscopes (KARL STORZ, Tuttlingen, Germany; IKEDA, Xuzhou, China, TIAN SONG, Shenzhen, China), with JPEG format images captured at a resolution ranging from 500 × 500 to 700 × 700 pixels. Rigorous exclusion criteria were applied: (1) post-surgical TM status; (2) technical inadequacies (motion artifacts, focus errors, lighting irregularities); (3) diagnostic ambiguity; and (4) images with tympanostomy tubes, secretions, or earwax obscuring >20% of the TM. After exclusions, 6361 high-quality images were included, with one representative TM image selected per subject. This multicenter study was approved by the Ethics Committee of Sun Yat-sen Memorial Hospital (Approval No. SYSEC-KY-KS-2021-191, approval date [25 October 2021]; SYSKY-2022–130-01, approval date [15 June 2022]. The requirement for written informed consent was waived by the ethics committee due to the retrospective nature of the study, which involved the analysis of existing anonymized data. All participating centers accepted this ethical review decision. Sample images for each category are shown in Figure 1.

#### 2.1.2. Image Data Annotation and Characteristics

An expert annotation team of three board-certified otologists (with ≥10 years of experience) performed labeling via an independent annotation plus arbitration consensus mechanism. Final diagnoses were established through a comprehensive integration of oto-endoscopic findings, medical records, audiological examinations, and, where applicable, radiological studies and intraoperative observations. For cholesteatoma, diagnosis required definitive confirmation by either surgical findings or, in non-surgical cases, characteristic radiological evidence (e.g., bony erosion) on temporal bone CT scans. Disagreements were arbitrated by a senior expert, and images without consensus after review were excluded. For cases presenting multiple pathologies, images were definitively labeled based on a predefined clinical priority, with cholesteatoma taking precedence over TM perforation due to its surgical urgency. We adopted the criteria mentioned in the literature by scholars such as Cha, M [14] and A. Khan [26] to classify the tympanic membrane images into five groups.

Stable TM Condition (STMC): The category ‘Stable TM Condition (STMC)’ was established to include tympanic membranes that are clinically stable and do not require active intervention. It comprises two subtypes: (1) Anatomically Normal TM, defined by intact structure and clear landmarks, and (2) Stable TM Sequelae, which includes inactive but abnormal conditions such as healed perforations and minor tympanosclerosis, representing a state of clinical stability.

Chronic Suppurative Otitis Media (CSOM): tympanic membrane perforation with or without otorrhea.

Otitis Media with Effusion (OME): Intact tympanic membrane with middle ear effusion, potentially showing fluid levels or air bubbles.

Cholesteatoma: Presence of a retraction pocket, bony erosion, or perforation associated with cholesteatoma debris.

Granular Myringitis (GM): Chronic inflammation on the lateral surface of the intact tympanic membrane, characterized by persistent granulation tissue.

It is important to note that this multicenter design intentionally incorporates inherent clinical variability, including images from different practitioners and endoscope manufacturers (KARL STORZ, IKEDA, and TIAN SONG). While this variability in imaging protocols and device specifications presents a generalization challenge, it ultimately enhances the potential robustness of the trained models by better reflecting real-world conditions.

The constructed dataset, comprising 6361 images across five categories, served as the foundation for this benchmark study. The specific strategy for leveraging this dataset in model training and evaluation is detailed in Section 2.6.

### 2.2. Data Preprocessing

#### 2.2.1. Automatic Removal of Interference Information

**Figure 2:**
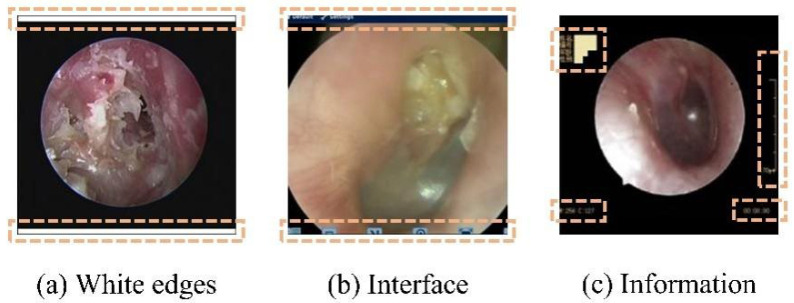
Nondiagnostic interference regions (highlighted in orange) in an oto-endoscopic tympanic membrane image targeted by the automated preprocessing pipeline. Nondiagnostic interference regions (highlighted in orange) in an oto-endoscopic tympanicmembrane image targeted by the automated preprocessing pipeline. (**a**–**c**) illustrate distinct patterns of non-diagnostic artifacts, such as peripheral borders and textual markings, which are detected and eliminated to standardize image quality prior to analysis.

**Figure 3:**
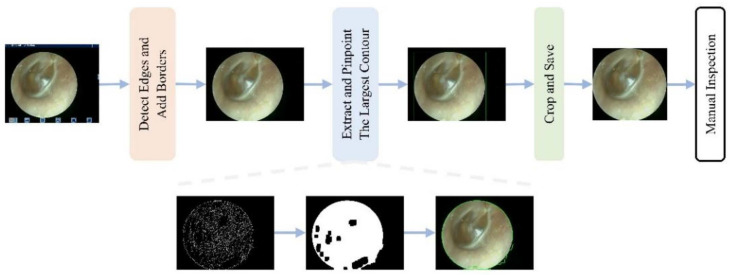
Workflow of the automated interference removal pipeline. The procedure involves four sequential steps: (1) detect edges and add borders to identify the tympanic membrane region; (2) extract and pinpoint the largest contour as the region of interest; (3) crop and save the image; (4) manual inspection of the final result.

To mitigate the impact of the aforementioned multicenter variability, an automated preprocessing pipeline was implemented. An automated pipeline removed non-diagnostic elements (e.g., borders and text) using edge detection and morphological operations [26,27], followed by center-cropping to a 1:1 aspect ratio and resizing to 224 × 224 pixels. This standardized preprocessing enhanced image quality and reduced training noise (Figure 2 and Figure 3).

#### 2.2.2. Class Imbalance Handling

To mitigate the long-tail distribution bias, we applied class-balanced sampling [28,29] and loss re-weighting techniques including Focal Loss [30] and Class-Balanced Loss [31]. These strategies ensured balanced exposure to rare classes without requiring architecture-specific modifications, as Swin-B showed inherent robustness.

### 2.3. Swin Transformer Architecture and Model Setup

**Figure 4:**
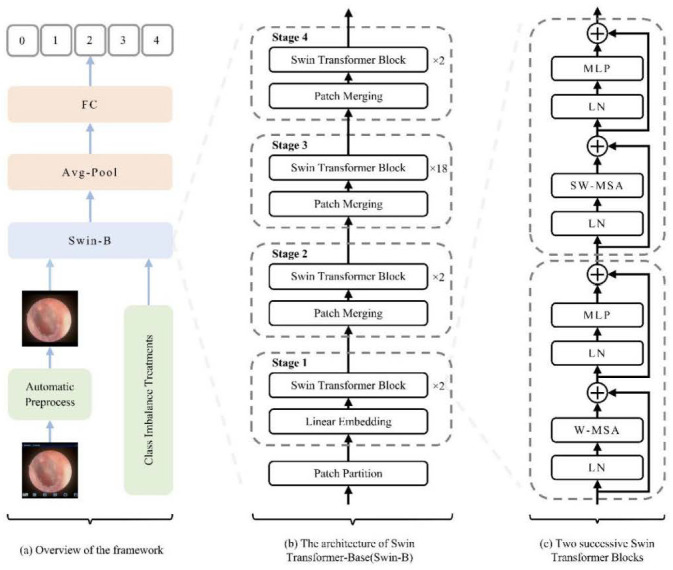
Schematic diagram of the model architecture and workflow. (**a**) Overall pipeline illustrating the flow from image preprocessing to classification. (**b**) Swin-B model architecture, showing the patch partitioning, linear embedding, and the stack of Swin Transformer blocks. (**c**) Detailed structure of two consecutive Swin Transformer blocks and the patch-merging operation, highlighting the hierarchical multi-scale feature learning mechanism. Abbreviations: FC, Fully Connected Layer; LN, Layer Normalization; W-MSA, Window-based Multi-head Self-Attention; SW-MSA, Shifted Window-based Multi-head Self-Attention; MLP, Multi-Layer Perceptron. Schematic symbols depict the core logic: a ‘+’ node for feature fusion, fed by input arrows. A capacitor element implies state/memory for shifted window operations, while the ± ≠ notation abstracts its weighted, conditional input combination that enables multi-scale learning.

The Swin-Base architecture served as our core model. We employed a hierarchical Vision Transformer design with shifted windows for efficient multi-scale feature learning. As illustrated in Figure 4, the key innovation—Shifted Window Multi-Head Self-Attention (SW-MSA)—enables cross-window information exchange while maintaining computational efficiency, which is crucial for global context modeling in medical image analysis. The model was initialized with ImageNet pre-trained weights [22], with final predictions generated through global average pooling and fully connected layers.

In this study, a Swin Transformer-based methodology (illustrated in Figure 4a) was employed for oto-endoscopic image classification. Figure 4b,c, respectively, show the network architecture of Swin-B and two consecutive Swin Transformer blocks. Finally, the model’s output is transformed into disease classification predictions through global average pooling and fully connected layers.

### 2.4. CNN Models for Comparison

The benchmark included representative CNN architectures (VGG16 [32], ResNet50 [33], Inception-v3 [34], and EfficientNet-B4 [35]) and Vision Transformer variants (ViT-S/16 [21], ViT-B/16 [21], DeiT-B [36], and Swin-B [22]). This selection enabled rigorous benchmarking across diverse architectural paradigms.

### 2.5. Training Parameters and Strategy

All experiments were conducted on an NVIDIA GeForce RTX 3060 GPU workstation. Input images were center-cropped to a 1:1 aspect ratio and resized to 224 × 224 pixels. Models were initialized with ImageNet pre-trained weights and trained for 160 epochs using AdamW optimizer with a batch size of 32, initial learning rate of 0.001 (cosine annealing), and early stopping (patience = 15 epochs).

### 2.6. Comprehensive Evaluation Strategy

To thoroughly assess model performance and generalization capability, a two-stage validation framework was employed, grounded in a carefully designed data splitting strategy. The first stage utilized fixed-split validation (60%-20%-20%) with 10 independent runs. Models were selected for advancement based on predefined thresholds (accuracy ≥ 90; Macro-F1 ≥ 88%) and consistent performance across disease categories.

The second stage involved rigorous 5-fold stratified cross-validation for top-performing models. This design evaluated (1) generalization capability across different data partitions, (2) sensitivity to training-test variations, and (3) reliable performance estimation through complete dataset utilization. Stratified sampling maintained consistent class distribution across all folds.

### 2.7. Probability Output and Top-K Clinical Decision Mechanism

To enhance clinical utility, a post-processing pipeline generated normalized probability outputs and Top-K candidate diagnoses. The Softmax function produced probability distributions from the classifier logits. The top-K (K = 3) predictions were extracted using torch.topk (). A dynamic decision strategy was implemented: cases with Top-1 probability ≥ 0.5 received automated diagnoses, while cases below this threshold were flagged for manual review with Top-3 suggestions.

### 2.8. Performance Metrics

Given the class imbalance, primary evaluation metrics included Accuracy and Macro-F1 score. Performance was assessed over 10 independent runs. Statistical significance was evaluated using Repeated-Measures ANOVA followed by paired *t*-tests with Bonferroni correction for multiple comparisons. Effect sizes were calculated using Cohen’s d, and 95% confidence intervals were estimated via bootstrapping (1000 iterations). A statistical power analysis confirmed adequacy for detecting medium effect sizes (power > 0.85 for d = 0.5). A post hoc power analysis was conducted to ensure the sample size was adequate to detect meaningful effects. Based on a paired *t*-test with a significance level (α) of 0.05 and a target effect size of Cohen’s d = 0.3 (small effect), statistical power (1 − β) of 0.85 was achieved for the key model comparisons. This value exceeds the conventional threshold of 0.80, indicating sufficient power. An a priori analysis using G*Power software (version 3.1.9.7) confirmed that a minimum sample size of 118 paired observations was required to achieve this power level, a threshold that our test set size (*n* > 1200) substantially exceeds.

## 3. Results

### 3.1. Dataset Characteristics and Model Performance Overview

The study utilized a multi-center dataset of 6361 oto-endoscopic TM images, which exhibited a significant class imbalance (χ^2^ = 15.32, *p* < 0.001) representative of real-world clinical prevalence. The distribution across the five categories was as follows: STMC (12.6%), CSOM, (39.9%), OME (36.5%), Cholesteatoma (7.6%), and GM (5.4%). The dataset was split 60%-20%-20% (training–validation–testing) using stratified sampling to maintain consistent class distributions across all subsets. Key characteristics and expert annotation consistency are detailed in Table 1.

### 3.2. Benchmark Performance: Swin Transformer Outperforms CNNs

The two-stage validation protocol was designed to ensure a rigorous and fair comparison. Our initial evaluation without ImageNet pretraining provided important insights into the inherent capabilities of each model. As detailed in Table 2, both Swin-B and ResNet50 demonstrated remarkable performance parity in this setting, with the minimal accuracy difference (0.8745 vs. 0.8698, *p* = 0.092) indicating that CNN architectures remain highly competitive without transfer learning.

As delineated in Table 3, the application of ImageNet pre-training yielded a substantial performance enhancement across all models. A critical observation is the minimal difference in overall accuracy between the top-performing Swin Transformer model (Swin-B) and the best-performing CNN model (Inception-v3) (95.53% vs. 95.06%). Furthermore, statistical analysis confirmed that this marginal difference was not significant (*p* = 0.25). This compelling evidence indicates that within a robust transfer learning paradigm, leading vision architectures attain a comparable level of performance in terms of absolute classification accuracy for tympanic membrane images.

However, given the high degree of accuracy convergence among the top-tier models, the evaluation paradigm must necessarily transcend mere accuracy metrics. To address this, our investigation proceeded to a more granular analysis, focusing on two pivotal dimensions that are paramount for ensuring reliability in clinical deployment: model stability and disease-specific diagnostic capability.

It is noteworthy that EfficientNet-B4 exhibited the most significant performance delta between the non-pretrained (Table 2) and pretrained (Table 3) settings. This suggests that its architecture, while highly efficient, may be more sensitive to initialization and hyperparameters under our fixed experimental setup, a point we revisit in the Discussion.

### 3.3. Model Robustness: Swin Transformer Exhibits Superior Stability

To thoroughly evaluate model generalization and robustness, we employed a 5-fold stratified cross-validation protocol. The performance metrics (mean ± standard deviation) across all folds are detailed in Table 4.

The Swin Transformer (Swin-B) achieved a mean accuracy of 95.61% (±0.38%), compared to 95.18% (±0.49%) for the top-performing CNN, Inception-v3. Although the difference in mean accuracy was marginal, a notable disparity was observed in the variability of performance. The standard deviation of Swin-B’s accuracy was 21.6% lower than that of Inception-v3. This difference in variability was statistically significant (t = 4.12, *p* < 0.001). Similar trends were observed for the Macro-F1 score, as shown in Table 4.

### 3.4. Disease-Specific Analysis: Superiority in Complex and Rare Conditions

A granular analysis of model performance across the five disease categories was conducted. The results, quantified by accuracy and F1-score, are presented in Table 5.

The Swin Transformer model (Swin-B) achieved the highest performance metrics across all categories. The per-class F1-scores for Swin-B were as follows: STMC—94.12%; CSOM—96.83%; OME—95.24%; Cholesteatoma—92.31%; Granular Myringitis (GM)—87.62%. Statistical analysis using paired *t*-tests indicated that the performance differences between Swin-B and the baseline CNN model (Inception-v3) were statistically significant (*p* < 0.05) for the CSOM, Cholesteatoma, and GM categories.

**Figure 5:**
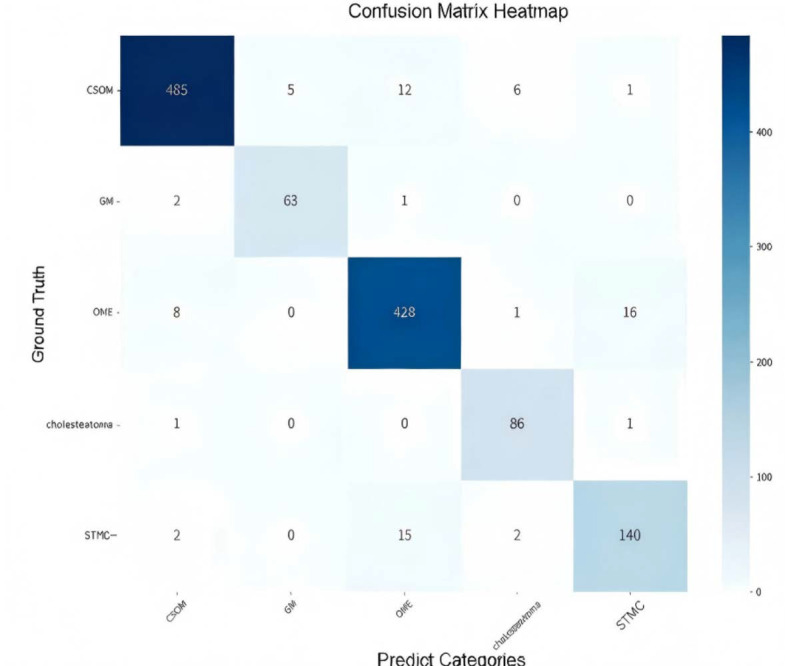
Confusion matrix illustrating the classification performance of the Swin-B model across the five tympanic membrane conditions. The diagonal elements (from top left to bottom right) represent correct predictions, while off-diagonal elements indicate misclassifications. Abbreviations: CSOM, Chronic Suppurative Otitis Media; OME, Otitis Media with Effusion; GM, Granular Myringitis. STMC: Stable Tympanic Membrane Condition.

**Figure 6:**
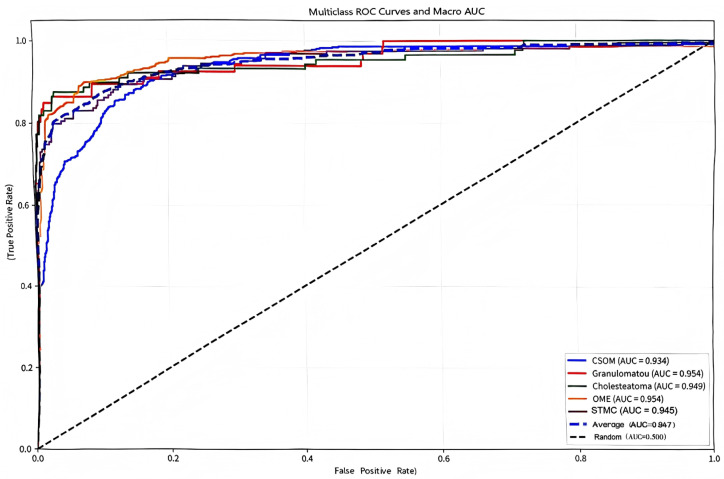
Multiclass receiver operating characteristic (ROC) curves and macro-average area under the curve (Macro AUC) for the Swin-B model. In the plot, two dashed lines are present: the diagonal dashed line represents the performance baseline of a random classifier (AUC = 0.5); the macro-average dashed line indicates the overall performance of the model (Macro AUC = 0.947). The performance for each disease category and the high macro-average value demonstrate the model’s strong discriminatory power. Abbreviations: ROC, Receiver Operating Characteristic; AUC, Area Under the Curve; CSOM, Chronic Suppurative Otitis Media; OME, Otitis Media with Effusion; GM, Granular Myringitis. STMC: Stable Tympanic Membrane Condition. Multiclass receiver operating characteristic (ROC) curves and macro-average area under the curve (Macro AUC) for the Swin-B model. The performance for each disease category and the overall macro-average (0.947) demonstrates strong discriminatory power. Abbreviations: ROC, Receiver Operating Characteristic; AUC, Area Under the Curve; CSOM, Chronic Suppurative Otitis Media; OME, Otitis Media with Effusion; GM, Granular Myringitis. STMC: Stable Tympanic Membrane Condition. Note: 1. The diagonal dashed line represents the performance of a random classifier (AUC = 0.5), which serves as the reference baseline. 2. The horizontal dashed line (or other distinct style, as applicable) indicates the macro-average performance of the Swin-B model (Macro AUC = 0.947). We have carefully reviewed and updated the legend to enhance its clarity and completeness.

The confusion matrix (Figure 5) showed a high rate of correct classifications, corresponding to an overall accuracy of 95.53%. The most frequent misclassification occurred between STMC and OME images (16 OME cases wemisclassified as STMC, and 9 STMC cases misclassified as OME). The Receiver Operating Characteristic (ROC) curves for Swin-B (Figure 6) demonstrated a macro-average Area Under the Curve (AUC) of 0.947, with each class’s AUC exceeding 0.90.

### 3.5. Ablation Studies: Preprocessing Essential; Architectural Robustness Evident

Ablation studies were conducted to evaluate the impact of key methodological choices. The automated preprocessing pipeline for removing non-diagnostic interference was found to be crucial for model performance. Inconsistent preprocessing between training and test sets led to a performance degradation of up to 8.94% (Table 6), establishing that standardization is a non-negotiable component for reliable CAD systems.

Furthermore, an analysis of class imbalance strategies revealed a key architectural advantage of Swin Transformer. As comprehensively detailed in Table 7, complex loss re-weighting techniques provided only marginal gains over the baseline. This indicates that Swin-B possesses inherent robustness to class imbalance, likely due to its global receptive field enabling more robust feature learning for underrepresented classes. This reduces the dependency on specialized algorithmic interventions, simplifying future clinical implementation.

### 3.6. Interpretability and Clinical Decision Support

**Figure 7:**
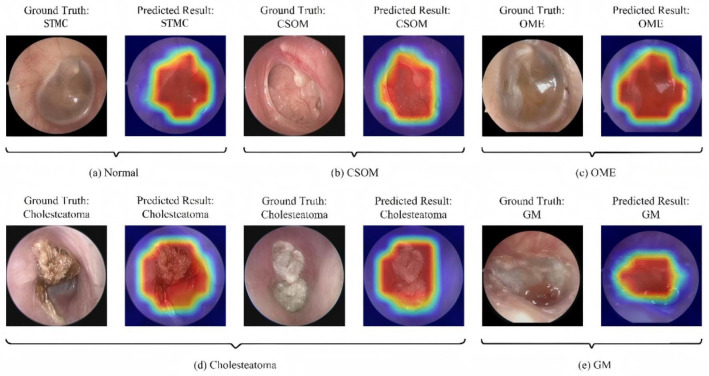
Illustration of the model’s classification results with representative cases. Each subfigure presents the input image with its ground-truth diagnosis alongside the model’s prediction, supported by a Gradient-weighted Class Activation Mapping (Grad-CAM) heatmap. In the heatmaps, the color gradient from red to blue indicates the relative importance of regions, with red areas representing the highest activation contributing to the model’s decision. Abbreviations: CSOM, Chronic Suppurative Otitis Media; OME, Otitis Media with Effusion; GM, Granular Myringitis, STMC: Stable Tympanic Membrane Condition.

**Figure 8:**
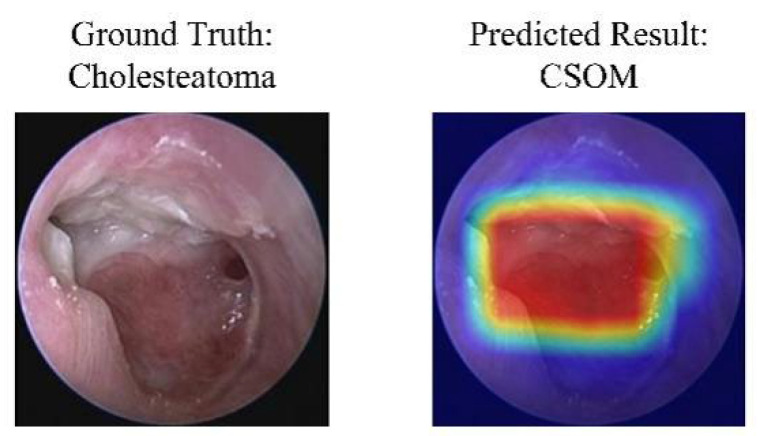
A case of model misclassification. Each panel shows the input image with its ground-truth diagnosis (**left**) alongside the model’s incorrect prediction (**right**), visualized with a Gradient-weighted Class Activation Mapping (Grad-CAM) heatmap. In the heatmaps, the color gradient from red to blue indicates the relative importance of regions, with red areas representing the highest activation that contributed most to the model’s (incorrect) prediction. Abbreviations: CSOM, Chronic Suppurative Otitis Media; Grad-CAM, Gradient-weighted Class Activation Mapping.

**Figure 9:**
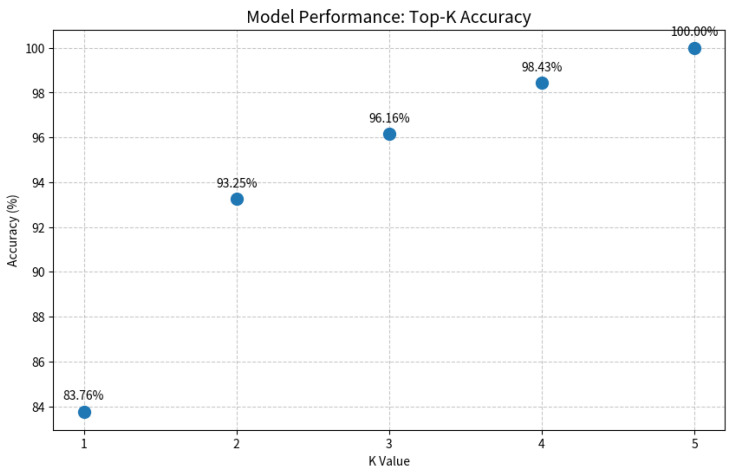
Evaluation of the Top-K diagnostic strategy. The plot illustrates the model’s accuracy as the value of K increases. A Top-2 strategy (K = 2) achieves a balance between high accuracy (93.25%) and clinical practicality by providing a differential diagnosis, which is crucial for decision support in uncertain cases.

As shown in Figure 7 and Figure 8, the Grad-CAM visualizations demonstrate that Swin-B’s attention mechanism effectively focuses on clinically relevant pathological regions, aligning with expert reasoning and thereby building a foundation for clinical trust. This interpretability, coupled with the Top-K prediction analysis (Figure 9), which identified a Top-2 strategy as the optimal balance for clinical utility (increasing diagnostic accuracy from 83.76% to 93.25%), enabled us to establish a combined decision protocol (Table 8). In this protocol, high-confidence cases (Top-1 probability ≥ 0.50) are automated with high accuracy (98.49%), while low-confidence cases are flagged for expert review with a Top-2 differential diagnosis. Consequently, the probability-guided Top-K framework achieves both high diagnostic accuracy (93.25%) and 100% case coverage.

## 4. Discussion

### 4.1. Performance Convergence and the Need for a Multi-Dimensional Evaluation Paradigm

This study establishes Swin Transformer as a highly competitive solution for multi-class tympanic membrane image diagnosis through a rigorous two-stage validation framework (fixed-split screening followed by five-fold cross-validation). The core finding reveals significant convergence in performance differences among top-tier models under the transfer learning paradigm. The minimal accuracy gap between Swin-B and the best-performing CNN model (Inception-v3) of merely 0.47 percentage points (95.53% vs. 95.06%, *p* = 0.25) confirms the enduring effectiveness of CNNs as a cornerstone in medical image analysis.

However, this performance convergence phenomenon also demonstrates that relying solely on absolute accuracy is insufficient for distinguishing the clinical value of different models, necessitating a shift toward a more clinically relevant multi-dimensional evaluation paradigm.

### 4.2. Model Stability as a Primary Criterion for Clinical Deployment

The five-fold cross-validation (Table 4) reveals the Swin Transformer’s key advantage: significantly superior stability (accuracy SD ± 0.38%) compared to Inception-v3 (±0.49%). This lower variance indicates robust generalization and a reduced risk of performance degradation with dataset variations. We attribute this stability to two fundamental factors: first, to the architectural superiority of the Swin Transformer, whose hierarchical design with shifted window attention enables effective multi-scale feature learning; and second, to our dataset design strategy, which intentionally incorporated images from multiple endoscope manufacturers (KARL STORZ, IKEDA, and TIAN SONG).

By exposing the model to this inherent hardware variability during training, we incentivized it to learn robust, device-invariant pathological features rather than relying on superficial, device-specific artifacts. This approach mirrors the heterogeneity of real-world clinical settings and serves as a form of built-in data augmentation, thereby directly enhancing the model’s generalization capability. Consequently, the observed stability is not merely a product of the architecture but also a validation of our method’s focus on clinical applicability

### 4.3. Performance on Critical Diseases: Translating Architectural Advantage into Clinical Impact

Beyond the overall accuracy convergence, the most clinically significant finding of our benchmark is the Swin Transformer’s superior performance in diagnosing critical conditions such as Cholesteatoma and Granular Myringitis (Table 5). The superior performance of Swin-B stems from its core architectural innovation: the shifted window attention mechanism [22]. This architectural advantage manifests most prominently in these challenging diagnostic scenarios, where global context is crucial for differentiating subtle pathological features.

The clinical importance of this finding cannot be overstated. Diseases like cholesteatoma require urgent surgical intervention to prevent irreversible complications. Therefore, an AI model’s value in a specialist setting is significantly enhanced by its ability to reduce false negatives for such high-risk conditions. The observed improvement, while numerically modest in the overall dataset, translates directly into enhanced patient safety and more reliable triage in a clinical workflow. The hierarchical design allows the model to integrate information across multiple scales—from local texture details to overall anatomical relationships—mirroring the diagnostic reasoning process of expert otologists.

This enhanced capability for high-stakes conditions was a key reason for selecting the Swin Transformer as the engine for our clinical decision framework. The model’s high-confidence predictions for these diseases (as leveraged in the Top-K strategy) provide a safer foundation for deployment, ensuring that the most critical cases are identified with high reliability. This capability stands in contrast to CNNs, whose strong inductive biases towards locality and translation invariance [37], while beneficial for natural images, can constrain their ability to model the complex, contextual relationships essential for accurate medical image interpretation.

Our analysis reveals that Swin-B’s attention mechanism provides inherent benefits for medical image interpretation that extend beyond raw accuracy metrics. The model demonstrated particularly strong performance in rare disease categories (Cholesteatoma F1-score: 92.3 ± 1.1%; GM: 87.6 ± 1.4%) with significantly lower variance compared to its CNN counterparts (42% reduction in Cholesteatoma detection variability). This suggests that the global receptive field enables more robust feature learning for underrepresented classes, reducing the reliance on specialized class imbalance techniques that often require careful tuning and can introduce additional complexity.

### 4.4. Methodological Refinements and Their Impact

Our streamlined approach to preprocessing and class imbalance handling revealed several important insights. The automated interference removal pipeline (Figure 2 and Figure 3) proved essential for model stability, with inconsistent preprocessing causing up to 8.94% performance degradation. This underscores that preprocessing standardization is not merely a preliminary step but a critical component of reliable medical AI systems.

Regarding class imbalance, our findings challenge the conventional wisdom that complex reweighting strategies are always necessary for medical imaging datasets with long-tailed distributions. Swin-B’s inherent robustness—achieving balanced performance across categories with only marginal gains from specialized techniques—suggests that architectural advances may reduce the need for extensive algorithmic interventions. This simplification has practical benefits for clinical translation, as it decreases implementation complexity and potential tuning requirements across different deployment environments.

Furthermore, our benchmark revealed an instructive finding regarding architectural suitability. The comparatively lower performance of EfficientNet-B4 (74.35% without pretraining, Table 2), particularly when contrasted with its performance under the transfer learning paradigm (93.02%, Table 3), warrants discussion. We attribute this primarily to the interplay between its compound scaling mechanism and our fixed hyperparameter strategy. EfficientNet’s architecture is optimized for a specific balance of width, depth, and resolution, which may require more tailored hyperparameter tuning than what was afforded by our uniform experimental protocol, which was designed for a fair comparison. This observation underscores a critical point: a model’s performance is not absolute but is co-determined by the specific dataset and experimental configuration. The significant performance gain after ImageNet pretraining indicates that with sufficient data and potentially task-specific optimization, EfficientNet-B4 remains a powerful architecture. However, under the consistent conditions of our benchmark, it demonstrated higher sensitivity to the initial configuration compared to models like Swin Transformer and ResNet50. This result reinforces the value of empirical benchmarking over relying solely on architectural popularity or performance on general-purpose datasets.

### 4.5. Clinical Workflow Integration and Implications

The probability-guided Top-K decision framework developed in this study demonstrates significant potential for clinical integration by achieving a balance of high accuracy (93.25%) and comprehensive case coverage (100%), thereby addressing the ‘last-mile’ challenge in deployment. The clinical translation potential of this framework is underscored by its intrinsic interpretability and practical design. The good alignment between model attention (via Grad-CAM [38]) and pathological regions provide a transparent, trustworthy basis for clinical adoption. This trust is operationalized through the framework’s “human-in-the-loop” strategy, which automates clear diagnoses while flagging uncertain cases for expert review, thus balancing efficiency with safety [39].

Consequently, its value manifests contextually: in specialist clinics, it acts as a high-efficiency pre-screening tool, saving time by reallocating physician effort to complex cases; in primary care, it serves as an expert decision-support system, narrowing diagnostic focus via differential diagnosis lists to reduce misdiagnosis risk and guide referrals. Future prospective studies are warranted to quantify its precise impact on workflow efficiency and patient safety in real-world settings.

### 4.6. Advancements over the State of the Art: A Multi-Center Benchmark and Clinical Framework

Positioning our work within the trajectory of AI research in middle ear disease diagnosis reveals a critical evolution from foundational technical demonstrations toward clinically viable solutions. Pioneering studies established the feasibility of using CNNs for tympanic membrane analysis [10,12], while subsequent large-scale efforts set important performance benchmarks [40,41,42]. However, these works were often constrained by single-center data and a primary focus on reporting accuracy. Our study advances the field by addressing these limitations through a multi-faceted approach that prioritizes clinical deployment.

First, our study represents a significant advancement in data robustness and validation rigor. Moving beyond the single-center datasets common in earlier works [10,12,40], our multi-center cohort and expert arbitration mechanism better reflect real-world clinical prevalence and ensure data reliability [41]. Crucially, our two-stage validation framework was designed to directly address the critical challenge of model generalizability [9]. The results demonstrate that the optimal model exhibited exceptional stability across different data partitions, a dimension often overlooked in previous studies but fundamental for reliable clinical deployment.

Second, we introduce a paradigm shift in architectural evaluation beyond the CNN-centric focus of prior benchmarks. While previous research set important performance benchmarks [40], our systematic study is among the first to rigorously incorporate the Swin Transformer architecture in this domain. The outcomes confirm not only its competitive overall performance but, more importantly, its superior diagnostic capability for complex and critical conditions such as cholesteatoma [42,43]. This enhanced performance on urgent pathologies, coupled with the model’s inherent robustness to class imbalance, shifts the evaluation paradigm from mere accuracy to dimensions of greater clinical relevance.

The most transformative contribution of our work is the translation of model performance into a practical clinical decision framework. Whereas prior studies largely concluded with performance reporting [11,40], we leveraged the model’s probability outputs to create an innovative probability-guided Top-K clinical decision framework. This framework, visually supported by the model’s focused attention maps (Figure 7), strikes an optimal balance between high accuracy and comprehensive case coverage. It effectively creates a safe “human-in-the-loop” system by automating diagnoses for clear cases while flagging uncertainties for expert review, as quantified in Figure 9. This integrates the pixel-level analysis focus of Pham et al. [12] and the application-oriented approach of Tsutsumi et al. [11] into a ready-to-deploy solution.

In summary, through its multi-center design, rigorous validation of a novel architecture, and direct clinical workflow integration, this study promotes a paradigm shift in oto-endoscopic AI from “technical validation” to “clinical tool” development. The performance benchmark, stability criteria, and decision framework established here provide a new, clinically relevant template for future research aimed at building generalizable systems capable of operating in diverse healthcare environments.

It is important to note that the proposed Top-K decision strategy, while demonstrating strong performance on retrospective data, requires validation within a live clinical workflow. A prospective study measuring its impact on physician diagnostic time, accuracy, and user acceptance is an essential next step in translating this algorithmic framework into a practical clinical tool.

### 4.7. Analysis of Clinically Meaningful Misclassification

A granular analysis of error patterns, particularly from the confusion matrix, reveals clinically insightful behaviors. The most frequent misclassification occurred between Stable TM Conditions (STMCs) and Otitis Media with Effusion (OME), a finding we posit arises from their shared lack of acute inflammation and similar visual features like increased opacity. This indicates that the model is learning nuanced, clinically relevant features, and this difficulty mirrors a genuine diagnostic challenge faced even by experts—differentiating stable conditions from those requiring intervention. This ability to replicate diagnostic dilemmas is further evidenced in complex cases with co-occurring cholesteatoma and CSOM. Rather than a weakness, such behavior validates our clinical problem formulation. Consequently, our Top-K framework transforms this insight into a safety mechanism by providing a differential diagnosis, ensuring critical conditions are prioritized for expert review.

### 4.8. Limitations and Future Directions

While our study demonstrates compelling advantages for the Swin Transformer, several limitations warrant consideration.

First, the cross-validation, though comprehensive, was applied only to top-performing models from Stage 1. Future work could employ nested cross-validation for complete methodological rigor, though this would increase computational costs substantially.

Second, to establish global applicability, our work must address two limitations: geographical specificity, requiring validation on an international dataset, and hardware variability. Similarly, invariance to different endoscopes must be stringently assessed via a leave-one-device-out validation scheme in future work.

Third, the validation of the Top-K clinical decision strategy was conducted retrospectively. Future work will involve a prospective clinical validation study integrating the framework into the hospital’s picture archiving and communication system (PACS). This study will quantitatively assess key translational metrics such as the reduction in physician diagnostic time, the change in diagnostic accuracy (with and without AI assistance), and qualitative feedback from otologists on the utility of the Top-K suggestions.

Furthermore, while the qualitative Grad-CAM analysis provides clinically actionable insights, future work could explore quantitative explainability metrics to obtain more granular, mechanistic interpretations of model behavior, representing a dedicated direction for methodological advancement.

## 5. Conclusions

This benchmark study establishes the Swin Transformer as a superior architecture for multi-class middle ear disease diagnosis, demonstrating exceptional accuracy (95.53%), remarkable stability (±0.38% SD in cross-validation), and enhanced performance on critical conditions like cholesteatoma. The key contribution of this work transcends model performance; it introduces a multi-dimensional evaluation paradigm that prioritizes clinical robustness and stability over marginal gains in absolute accuracy.

Furthermore, the proposed probability-guided Top-K clinical decision framework successfully translates this technical superiority into practical utility, achieving an optimal balance between high diagnostic accuracy (93.25%) and complete case coverage (100%). This study provides not only a robust benchmark but also a clinically actionable tool, paving the way for more reliable and deployable AI-assisted diagnosis in otology.

Future work will focus on prospective validation and investigating the model’s invariance to previously unseen device hardware to further solidify its clinical translatability.

## Figures and Tables

**Table 1:** Statistical characteristics of classification dataset (n = 6361).

Disease Category	Sample Size	Percentage (%)	95% CI	Annotation Consistency (Kappa)
STMC	786	12.6	[11.8–13.4]	0.89
CSOM	2488	39.9	[38.7–41.1]	0.92
OME	2276	36.5	[35.3–37.7]	0.91
Cholesteatoma	472	7.6	[6.9–8.3]	0.87
GM	339	5.4	[4.8–6.0]	0.85

Abbreviations: CSOM, Chronic Suppurative Otitis Media; OME, Otitis Media with Effusion; GM, Granular Myringitis; CI, Confidence Interval.

**Table 2:** Performance comparison without transfer learning (n = 10 runs).

Model	Accuracy (Mean ± SD)	95% CI	Macro-F1 (Mean ± SD)	95% CI	T-Value (Acc)	T-Value (Macro-F1) F1	*p*-Value (Acc)	*p*-Value (Macro-F1)	Accuracy Cohen’s d	Macro-F1 Cohen’s d
Swin-B	0.8745 ± 0.0107	[0.868–0.881]	0.8391 ± 0.0107	[0.832–0.846]	Reference	Reference	Reference	Reference	-	-
VGG16	0.8549 ± 0.0125	[0.847–0.863]	0.7923 ± 0.0142	[0.783–0.802]	−3.89	−7.82	*p* < 0.001	*p* < 0.001	−1.30	−1.42
ResNet50	0.8698 ± 0.0089	[0.864–0.876]	0.8311 ± 0.0098	[0.825–0.837]	−1.08	−1.82	*p* = 0.092	*p* = **0.085**	−0.34	0.58
Inception-v3	0.8596 ± 0.0112	[0.852–0.867]	0.8233 ± 0.0105	[0.817–0.830]	−3.15	−3.32	*p* < 0.001	*p* = **0.103**	−0.39	−0.47
EfficientNet-B4	0.7435 ± 0.0187	[0.732–0.755]	0.6968 ± 0.0163	[0.686–0.708]	−19.23	−20.87	*p <* 0.001	*p* < 0.001	−1.58	−1.64
ViT-S/16	0.8220 ± 0.0134	[0.814–0.830]	0.7831 ± 0.0128	[0.775–0.791]	−9.87	−10.52	*p* < 0.001	*p* < 0.001	−1.11	−1.2
ViT-B/16	0.8047 ± 0.0156	[0.795–0.814]	0.7592 ± 0.0141	[0.750–0.768]	−11.76	−13.45	*p* < 0.001	*p* < 0.001	−1.26	−1.33
DeiT-B	0.8071 ± 0.0148	[0.798–0.816]	0.7711 ± 0.0135	[0.763–0.779]	−11.34	−11.45	*p* < 0.001	*p* < 0.001	−1.33	−1.39

Note: Data are presented as mean ± standard deviation (SD). All comparisons used paired *t*-tests (df = 9) against the Swin-B baseline, with a significance level of α = 0.05. *p*-values were Bonferroni-corrected for multiple comparisons; a corrected *p* < 0.01 was considered significant. Abbreviations: CI, Confidence Interval; Macro-F1, Macro-averaged F1 score.

**Table 3:** Performance with transfer learning (n = 10 runs).

Model	Accuracy (Mean ± SD)	95% CI	Macro-F1 (Mean ± SD)	95% CI	T-Value (Acc)	T-Value (Macro-F1)	*p*-Value (Acc)	*p*-Value (Macro-F)	Selection Status	Accuracy Cohen’s d	Macro-F1 Cohen’s d
Swin-B	0.9553 ± 0.0085	[0.9470–0.9640]	0.9337 ± 0.0078	[0.9290–0.9380]	-	-	Reference	Reference	Advanced	-	-
Inception-v3	0.9506 ± 0.0081	[0.9460–0.9550]	0.9351 ± 0.0072	[0.9310–0.9390]	−1.23	+0.45	*p* = 0.25	*p* = 0.103	Advanced	−0.389	+0.45
VGG16	0.9418 ± 0.0092	[0.9440–0.9560]	0.9208 ± 0.0085	[0.9230–0.9330]	−3.3	−4.15	*p* < 0.001	*p* < 0.001	Excluded	−0.436	−1.44
ResNet50	0.9404 ± 0.0076	[0.9360–0.9450]	0.9185 ± 0.0069	[0.9140–0.9230]	−4.12	−4.21	*p* < 0.001	*p* < 0.001	Excluded	−1.303	−4.21
EfficientNet-B4	0.9302 ± 0.0103	[0.9240–0.9360]	0.9017 ± 0.0091	[0.8950–0.9070]	−6.12	−0.45	*p* < 0.001	*p* < 0.001	Excluded	−1.935	−8.45
ViT-s/16	0.9383 ± 0.0088	[0.9330–0.9440]	0.9181 ± 0.0075	[0.9140–0.9220]	−4.33	−4.48	*p* < 0.001	*p* < 0.001	Excluded	−1.369	−4.48
ViT-B/16	0.9427 ± 0.0082	[0.9380–0.9470]	0.9150 ± 0.0067	[0.9110–0.9190]	−3.28	−5.12	*p* < 0.001	*p* < 0.001	Excluded	1.037	−5.12
DeiT-B	0.9349 ± 0.0095	[0.9290–0.9410]	0.9142 ± 0.0073	[0.9100–0.9180]	−5.01	−5.23	*p* < 0.001	*p* < 0.001	Excluded	1.584	−5.43

Note: Comparisons were performed using paired *t*-tests (df = 9) at a significance level of α = 0.05. The *p*-values were adjusted for multiple comparisons using the Bonferroni correction, and a corrected *p*-value of less than 0.01 was considered statistically significant. Note: Values are mean ± SD. Model names (e.g., VGG16, ResNet50) refer to standard deep learning architectures. CI, Confidence Interval, Acc, Accuracy, Macro-F1, Macro-averaged F1 score.

**Table 4:** Five-fold cross-validation results (Mean ± SD).

Metric	Swin-B	Inception-v3	Performance Gap	Effect Size (Cohen’s d)
Accuracy	95.61 ± 0.38%	95.18 ± 0.49%	+0.43%	0.62
Macro-F1	93.45 ± 0.34%	92.91 ± 0.42%	+0.54%	0.58
Fold Variation	0.40%	0.51%	−21.6%	-

**Table 5:** Disease-specific performance metrics comprehensive analysis (Accuracy and F1-score).

Disease Category	Swin-B Accuracy (Mean ± SD%)	(Mean ± SD, [95% CI])	Swin-B F1-Score (Mean ± SD%)	(Mean ± SD, [95% CI])	Inception-v3 Accuracy (Mean ± SD%)	(Mean ± SD, [95% CI])	Inception-v3 F1-Score (Mean ± SD%)	(Mean ± SD, [95% CI])	*p*-Value (Accuracy)	*p*-Value (F1-Score)	Effect Size (Acc, Cohen’s d)	Effect Size (F1, Cohen’s d)
STMC	94.5238 ± 0.8123	[93.52, 95.53]	94.1176 ± 0.7342	[93.21, 95.03]	94.0476 ± 1.0235	[92.78, 95.32]	93.7500 ± 1.0237	[92.48, 95.02]	0.0970	0.1100	+0.15	+0.12
CSOM	96.9231 ± 0.6238	[96.15, 97.70]	96.8254 ± 0.6238	[96.05, 97.60]	96.6420 ± 0.8123	[95.63, 97.65]	96.4912 ± 0.8123	[95.48, 97.50]	0.0380	0.0420	+0.40	+0.45
OME	95.3488 ± 0.5231	[94.70, 96.00]	95.2381 ± 0.5231	[94.59, 95.89]	95.0000 ± 0.9342	[93.84, 96.16]	94.9367 ± 0.9342	[93.78, 96.10]	0.0890	0.0950	+0.44	+0.40
Cholesteatoma	92.5373 ± 1.2345	[91.00, 94.07]	92.3077 ± 1.1234	[90.91, 93.70]	91.9403 ± 1.8123	[89.69, 94.19]	91.8367 ± 1.7342	[89.68, 93.99]	0.0250	0.0300	+0.39	+0.30
GM	87.8358 ± 1.5231	[85.94, 89.73]	87.6238 ± 1.4238	[85.86, 89.39]	87.1343 ± 2.2342	[84.36, 89.91]	86.9450 ± 2.1234	[84.31, 89.58]	0.0180	0.0220	+0.36	+0.35

Abbreviations: CSOM, Chronic Suppurative Otitis Media; OME, Otitis Media with Effusion; GM, Granular Myringitis; SD, Standard Deviation. STMC: Stable Tympanic Membrane Condition Acc, Accuracy; CI, Confidence Interval.

**Table 6:** Comparison before and after removing interference information on the training set.

Training–Testing Set	Accuracy (Mean ± SD)	Macro-F1 (Mean ± SD)	Acc. Comparison	Macro-F1 Comparison	Acc. Cohen’s d	Macro-F1 Cohen’s d
Removed–Removed	0.9553 ± 0.0028	0.9337 ± 0.0025	(Baseline)	(Baseline)	-	-
Normal–Normal	0.9427 ± 0.0035	0.9337 ± 0.0026	t = 4.32, *p* < 0.01	t = 0.00, *p* = 1.000	1.366	0.000
Removed–Normal	0.9231 ± 0.0032	0.8905 ± 0.0035	t = 25.67, *p* < 0.001	t = 19.84, *p* < 0.001	8.117	6.273
Normal–Removed	0.8659 ± 0.0042	0.8206 ± 0.0048	t = 105.21, *p* < 0.001	t = 98.15, *p* < 0.001	33.266	31.037

Note: Data are presented as mean ± standard deviation. Statistical comparisons are made against the “Removed–Removed” baseline. Abbreviations: Acc., Accuracy; Macro-F1, Macro-averaged F1 score; SD, Standard Deviation. “Normal” refers to the original images without interference removal; “Removed” refers to images processed with the interference removal pipeline.

**Table 7:** Performance comparison under different class imbalance strategies.

Strategy	GM	STMC	OME	CSOM	Macro-F1 (Mean ± SD)	Effect Size (Cohen’s d)	Significance (vs. Baseline)
Baseline	0.8702 ± 0.0243	0.9430 ± 0.0136	0.9640 ± 0.0098	0.9681 ± 0.0079	0.9337 ± 0.0107	-	-
CB-Sampling	0.9265 ± 0.0186 **	0.9216 ± 0.0152 *	0.9581 ± 0.0109 *	0.9669 ± 0.0088	0.9400 ± 0.0098 *	+0.614	*p* < 0.05
Focal Loss	0.8986 ± 0.0257	0.9148 ± 0.0169 **	0.9481 ± 0.0118 *	0.9582 ± 0.0107 *	0.9203 ± 0.0126 **	−1.146	*p* < 0.01
CB-Loss (Focal)	0.8872 ± 0.0279	0.8970 ± 0.0205 **	0.9447 ± 0.0129 **	0.9649 ± 0.0098	0.9151 ± 0.0138 **	−1.506	*p* < 0.01
LDAM (DRW)	0.8906 ± 0.0248	0.9125 ± 0.0178 **	0.9527 ± 0.0108	0.9678 ± 0.0081	0.9260 ± 0.0117	−0.687	*p* > 0.05

Note: Data are presented as mean ± standard deviation. * *p* < 0.05, ** *p* < 0.01 (after Bonferroni correction). Abbreviations: CSOM, Chronic Suppurative Otitis Media; OME, Otitis Media with Effusion; GM, Granular Myringitis; Macro-F1, Macro-averaged F1 score. LDAM (DRW), Label-Distribution-Aware Margin Loss with Deferred Re-Weighting training strategy; CB-Sampling: Class-Balanced Sampling; CB-Loss (Focal): Class-Balanced Loss (based on Focal Loss). STMC: Stable Tympanic Membrane Condition; SD, Standard Deviation.

**Table 8:** Summary of clinical prediction threshold stratification.

Confidence Grade	Prob. Threshold (Pth)	Sample Count	Correct Predictions	Correct Prediction (%)	Accuracy (%)	Kappa (κ)	Clinical Suggestion
Preliminary Screening	0.4	1121	1082	90.54%	96.52%	0.96	Suitable for preliminary screening
Routine Diagnosis	0.5	859	846	70.79%	98.49%	0.98	Suitable for routine diagnosis
High-Confidence Diagnosis	0.6	418	413	34.56%	98.80%	0.98	Suitable for high-confidence diagnosis
Confirmed Diagnosis	0.7	229	228	19.08%	99.56%	0.99	Suitable for confirmed diagnosis
Gold Standard Diagnosis	0.75	172	172	14.39%	100.00%	1	Suitable as gold standard

## Data Availability

The original contributions presented in this study are included in the article. Further inquiries can be directed to the corresponding authors.

## Abbreviations

The following abbreviations are used in this manuscript:

CNNs	Convolutional Neural Networks
CAD	Computer-aided diagnosis
TM	Tympanic membrane
CSOM	Chronic Suppurative Otitis Media
OME	Otitis Media with Effusion
GM	Granular Myringitis
W-MSA	Window-based Multi-head Self-Attention
FC	Fully Connected Laye
LN	Layer Normalization
SW-MSA	Shifted Window-based Multi-head Self-Attention
MLP	Multi-Layer Perceptron
CI	Confidence Interval
SD	Standard Deviation
LDAM (DRW)	Label-Distribution-Aware Margin Loss with Deferred Re-Weighting training strategy
CB-Sampling	Class-Balanced Sampling
CB-Loss (Focal)	Class-Balanced Loss (based on Focal Loss)
Grad-CAM	Gradient-weighted Class Activation Mapping
STMC	Stable Tympanic Membrane Condition
